# Reducing Health Inequities Through Intersectoral Action: Balancing Equity in Health With Equity for Other Social Goods

**DOI:** 10.15171/ijhpm.2018.103

**Published:** 2018-10-28

**Authors:** Maxwell J. Smith, Daniel Weinstock

**Affiliations:** ^1^School of Health Studies, Faculty of Health Sciences, Western University, London, ON, Canada.; ^2^Institute for Health and Social Policy, McGill University, Montreal, QC, Canada.

**Keywords:** Health Equity, Health Inequities, Social Justice, Intersectoral, Ethics

## Abstract

Significant attention has been devoted to developing intersectoral strategies to reduce health inequities; however, these strategies have largely neglected to consider how equity in health ought to be weighted and balanced with the pursuit of equity for other social goods (eg, education equity). Research in this domain is crucial, as the health sector’s pursuit of health equity may be at odds with policies in other sectors, which may consider the reduction of health inequities to be peripheral to, if not incompatible with, their own equity-related aims. It is therefore critical that intersectoral strategies to reduce health inequities be guided by a more general account of social justice that is capable of carefully balancing equity in health against the pursuit of equity in other sectors.

## Background

Social policies outside of the health sector—in domains like education, finance, transportation, and housing—contribute to the background social conditions that shape the population’s health and mediate the effectiveness of health policy, healthcare, and public health interventions. Protecting and promoting population health therefore demands extensive knowledge of the interactions between health and social policy. In recognition of this important health and social policy nexus, the health sector (ie, that which includes healthcare, public health, and so forth) has increasingly sought to target and improve key social conditions by intervening in the social determinants of health and by introducing mechanisms that enable the systematic consideration of health implications in policy-making across government sectors—an approach variably referred to as ‘health in all policies’ or ‘healthy public policy.’^1^

Central to these initiatives is a recognition that disparities in the distribution of social conditions in a society can in turn create, sustain, and exacerbate health disparities between population groups. When health disparities are caused by disparities in social conditions that are perceived to be unjust or unfair—what would be considered ‘social injustices’—they are commonly referred to as ‘health inequities’ and are considered of great ethical importance to remediate.Accordingly, the reduction of health inequities has been placed at the top of public health agendas around the world, and tools specifically designed to assess the equity impacts of public health interventions, such as ‘health equity impact assessments,’ are increasingly being developed and implemented in public health policy and practice.^2^

## Intersectoral Action on Health Inequities

Despite these important initiatives, health inequities persist. Substantial and sustainable progress on health equity appears to be predicated on the willingness and capacity of non-health sectors to support and invest in this health goal.^3-5^ Thus, it is crucially important to explore how intersectoral collaboration to reduce health inequities can be realized.

Unsurprisingly, significant attention has been devoted to studying and developing intersectoral strategies for health equity.^6-9^ This includes important contributions that have focused on the political and ideological factors that may stymie intersectoral collaboration and, ultimately, progress in the pursuit of this aim.^10-13^ Yet, arguably, intersectoral strategies for health equity* by their very nature* may limit the opportunity or appetite of non-health sectors to collaborate. This is because intersectoral action on health inequities takes as its starting point the privileging of equity in *health* over equity for other social goods (otherwise it would be, for example, ‘intersectoral action on education inequities’ or, more generally, ‘intersectoral action on social inequities’). This ought to be considered problematic, as the health sector’s pursuit of *health* equity may ultimately be at odds with the pursuit of equity in other sectors, which may consider the reduction of health inequities to be peripheral to, if not incompatible with, their own context-specific equity objectives. For example, in the education sector (a central social determinant of health), practices aimed at the reduction of health disparities may understandably be overlooked, deprioritized, or undermined if they come at the cost (or are perceived to come at the cost) of the education system’s pursuit of equitable education processes and outcomes (ie, ‘education equity’). An education system that countenances parental school choice (ie, the option to enroll one’s child in schools other than those publicly provided, like private schools, charter schools, or faith-based schools), for instance, may result in different educational outcomes for children in each system,^14^ which could in turn lead to health disparities given correlations between education performance gaps and health outcomes.^15^ Yet, such a policy may still be defended on *education equity* grounds (or broader social justice grounds) if one subscribes to the notion that education equity entails allowing parents to choose where their children will be educated.^16^ On the other hand, though, there will certainly be instances where values and objectives in other sectors cohere with the pursuit of health equity, and it will be equally important to identify these instances of value congruence in order to promote intersectoral collaboration and policy synergy. For instance, the identification and elimination of discriminatory practices from schools and classrooms as a matter of education equity can work to narrow education performance gaps, which may work in turn to reduce health inequalities.

Furthermore, there may be reasons grounded in equity for governments to not interfere in non-health sectors even when policies in those sectors create health inequalities. As Weinstock argues, “were it to be demonstrated that aspects of the educational system impact upon health distributions, it would be a hasty inference to conclude that educational policy ought to be geared exclusively at the best health outcomes and at the most equitable distributions of health states. Merit, to name but one other normative criterion, ought not to be completely expunged from the determination of the distribution of certain educational resources” (pg. 82).^17^ When intervening in non-health sectors for the sake of health equity, health considerations must be balanced against other equity goals that are served by those sectors. In other words, due to the need to realize equity for social goods in addition to health, there will be instances where it would be inappropriate for governments to prioritize the achievement of *health* equity by acting upon the distribution of social determinants.

Without accounting for and ultimately striving to achieve congruence across government sectors with respect to how equity is to be pursued and how health considerations are to be balanced against social goals served by those sectors, intersectoral strategies to reduce health inequities may be inhibited or altogether thwarted. It is therefore critical that government-wide, intersectoral strategies to reduce health inequities understand and develop mechanisms to accommodate and address the unique and potentially conflicting aims and approaches to equity that exist between the health sector and other areas of social policy. The challenge lies in identifying the sectoral values and prescriptive standards underlying different sectors’ pursuit of equity and establishing a theoretical framework that can be used to assess the degree to which those values cohere to advance equity aims *across* sectors. It is to this framework that we now turn our attention.

## Public Health Ethics and the Role of Social Justice in “Intersectoriality”

The study of ethical values and issues in medicine and healthcare has been widely established and institutionalized over the past 50 years through research, scholarship, and practice in the field of bioethics. However, it was only recently that the field’s traditional focus on ethical issues involved in the treatment of individuals in medicine and healthcare was reoriented to systematically examine the distinctive moral questions that arise in the context of public health.

The most salient and significant area of ethical inquiry in the context of public health concerns the examination of which considerations or account of social justice ought to guide public health policy and practice, and indeed the pursuit of health equity.^18^ Generally speaking, theories of social justice concern themselves with delineating whether and where it is ethically appropriate for governments to wield their coercive powers in order to enforce the just distribution of particular goods, like healthcare, health, or income. They may also provide insights into whether, and the degree to which, principles and considerations of social justice ought to guide local conditions and contexts.

Theoretical discussions of social justice in the field of public health ethics have been rich, yet the translation or application of these theoretical insights to health policy and research and the intersectoral pursuit of health equity has been lacking.^19^ For instance, it appears that the introduction of health equity considerations into ‘health in all policies’ and ‘health equity impact assessments’ has perhaps taken for granted the significance of health and its just distribution relative to other social goods, and may have created the impression that governments should use their coercive powers to ensure the just distribution of health is enforced even if this creates disparities (and indeed, ‘inequities’) among other important social goods. The intersectoral pursuit of health equity therefore ought to be guided by an account of social justice that carefully weighs equity in health against the pursuit of the equitable distribution of social goods in other sectors. Many accounts of social justice are on offer which could further this aim, including those developed out of particular concern for questions of justice in health,^18,20-23^ yet additional work must be done to apply insights from such accounts in order to theoretically ground intersectoral strategies for health equity. Experiences exploring and pursuing intersectoral action on health inequities should similarly inform the ways in which we theorize the proper balancing of equity in health with the equitable distribution of other social goods.

## Conclusions

In sum, we argue that it is prudent to examine how the pursuit of health equity in the health sector aligns with the conceptualization and pursuit of equity in other sectors where ‘health in all policies’ approaches are being implemented or considered. In order to successfully (and ethically) pursue health equity intersectorally, we ought to investigate where conflict or coherence between these approaches exist, and explore opportunities to move toward a more comprehensive aim of social justice.

## Acknowledgements

Financial support for this work was provided to Maxwell J. Smith by a Canadian Institutes of Health Research Banting Postdoctoral Fellowship (#144487).

## Ethical issues

Not applicable.

## Competing interests

Authors declare that they have no competing interests.

## Authors’ contributions

Each author contributed equally to the conceptualization and writing of this manuscript. The manuscript has been read and approved by all authors.

## Authors’ affiliations

^1^School of Health Studies, Faculty of Health Sciences, Western University, London, ON, Canada. ^2^Institute for Health and Social Policy, McGill University, Montreal, QC, Canada.
